# Perioperative Serum Scoring Systems Predict Early Recurrence and Poor Prognosis of Resectable Pancreatic Cancer

**DOI:** 10.3389/fonc.2022.841819

**Published:** 2022-02-21

**Authors:** Shengnan Li, Gengpu Zhang, Yang Lu, Tiansuo Zhao, Chuntao Gao, Weishuai Liu, Yongjun Piao, Yanan Chen, Chongbiao Huang, Antao Chang, Jihui Hao

**Affiliations:** ^1^ Department of Pancreatic Cancer, Tianjin Medical University Cancer Institute and Hospital, National Clinical Research Center for Cancer, Key Laboratory of Cancer Prevention and Therapy, Tianjin’s Clinical Research Center for Cancer, Tianjin, China; ^2^ School of Medicine, Nankai University, Tianjin, China

**Keywords:** systemic immune inflammation, coagulation system, tumor marker, resectable pancreatic cancer, early recurrence, prognosis

## Abstract

**Objective:**

Some patients with pancreatic ductal adenocarcinoma (PDAC) are prone to rapid recurrence or metastasis after radical resection. However, evaluation methods for effectively identifying these patients are lacking. In this study, we established perioperative serum scoring systems to screen patients with early recurrence and poor prognosis.

**Methods:**

We systematically analysed 44 perioperative serum parameters, including systemic inflammatory parameters, coagulation system parameters, tumor markers, and 18 clinicopathological characteristics of 218 patients with radical resection in our centre. Univariate Cox regression and LASSO regression models were used to screen variables. Kaplan-Meier survival analysis was used to compare relapse-free survival and overall survival. Multivariate Cox regression was used to evaluate the independent risk variables. AUC and C-index were used to reveal the effectiveness of the models. In addition, the effectiveness was also verified in an independent cohort of 109 patients.

**Results:**

Preoperative systemic immune coagulation cascade (SICC) (including increased neutrophil to lymphocyte ratio, decreased lymphocyte to monocyte ratio, increased platelet and fibrinogen) and increased postoperative tumor markers (TMs) (CA199, CEA and CA242) were independent risk factors for early recurrence of resectable pancreatic cancer. On this basis, we established the preoperative SICC score and postoperative TMs score models. The patients with higher preoperative SICC or postoperative TMs score were more likely to have early relapse and worse prognosis. The nomogram based on preoperative SICC, postoperative TMs, CACI, smoking index, vascular cancer embolus and adjuvant chemotherapy can effectively evaluate the recurrence rate (AUC_1 year_: 0.763, AUC_2 year_: 0.679, AUC_3 year_: 0.657) and overall survival rate (AUC_1 year_: 0.770, AUC_3 year_: 0.804, AUC_5 year_: 0.763).

**Conclusion:**

Preoperative SICC and postoperative TMs can help identify resectable PDAC patients with early recurrence and poor prognosis.

## Introduction

Pancreatic ductal adenocarcinoma (PDAC) is one of the most lethal malignancies with a 5-year survival rate of 10%, mainly caused by insidious rapid recurrence or metastasis (1). Surgical resection is currently the most effective treatment for PDAC. However, only approximately 20% of patients have the opportunity to undergo radical resection at the time of diagnosis (2). Meanwhile, approximately half of these patients who underwent surgery can receive follow-up adjuvant chemotherapy (3, 4). Unfortunately, about a quarter of these patients will rapidly relapse within 6 months after surgery, but may benefit from neoadjuvant chemotherapy (5–7). Therefore, screening out these patients with high risk of early recurrence for precision medicine and early intervention could be an effective strategy to improve their prognosis and survival. However, there is no accessible evaluation system in clinic at present, driving us to establish new models for effective prediction of the early recurrence and overall survival (OS) of patients with resectable PDAC.

An increasing number of studies have shown that systemic immune inflammation amongst patients with cancer is closely related to their poor prognosis (8, 9). Neutrophils, lymphocytes, platelets, monocytes and the combinations of these factors, such as neutrophil-to-lymphocyte ratio (NLR), platelet-to-lymphocyte ratio (PLR) and lymphocyte-to-monocyte ratio (LMR), are associated with the prognosis of cancer patients (10–12). For example, lots of studies have demonstrated a causal relationship between neutrophils and metastasis (13–15). Meanwhile, it has been reported that the activation of platelets and the coagulation system also play essential roles in the progression of cancer (16). However, some studies have also reported that the proper use of these inflammatory parameters as prognostic factors is depended on the level of serum bilirubin (17). In addition, the intrinsic mechanisms of cancer cell heterogeneity between tumor-bearing hosts also largely determine metastasis driven by systemic immune inflammation (18). Hence, the effect of systemic immune inflammation on the prognosis of cancer patients may depend on the results of the comprehensive effects of the internal tumor microenvironment.

Currently, the most common tumor marker for PDAC in clinical practice is carbohydrate antigen 19-9 (CA19-9), but its usefulness in prognostic monitoring is limited because of low sensitivity and low specificity (19). In recent years, several pathological indexes, such as tumor size, tumor location, lymph node metastasis, TNM stage and pathological grade have been reported to possibly affect OS or disease recurrence of PDAC (20, 21). Meanwhile, serum index like serum alkaline phosphatase (ALP) in patients with early PDAC has also been reported to be significantly correlated with OS (22). However, the relationship between the baseline levels of other serum indexes, i.e., serum bilirubin, containing total bilirubin (TBIL) and direct bilirubin (DBIL), or albumin (ALB) and the prognosis of patients with PDAC remains controversial, and need to be further investigated (23, 24). Previous studies mainly focused on the limited parameters 1 month or more before or after surgery, whilst disregarding the influence of these indicators during the perioperative period, which in fact, is more crucial for predicting the prognosis of resectable PDAC (25). In this study, we evaluated the comprehensive effects of pathological indexes, systemic inflammation indicators, coagulation system parameters, tumor markers and other serum parameters on the early recurrence and the prognosis of resectable PDAC during the perioperative period. Our data indicated that preoperative systemic immune coagulation cascade (SICC) [including NLR, LMR, platelet and fibrinogen (Fbg)] and postoperative tumor markers (TMs) [CA19-9, carcinoembryonic antigen (CEA) and carbohydrate antigen 242 (CA242)] were pivotal in prediction of early recurrence and low survival of PDAC patients with radical resection. On this basis, we constructed a series of evaluation systems for effectively identifying PDAC patients with rapid recurrence or metastasis after radical resection, which may help clinicians make medical decisions and provide individualised treatment to patients with resectable PDAC.

## Materials and Methods

### Patients and Samples

This study recruited 327 PDAC patients with radical resection under the National Comprehensive Cancer Network (NCCN) guidelines from March 2012 to December 2018 at Tianjin Medical University Cancer Institute and Hospital (TJMUCIH) (26). All patients had intact abdominal CT or MRI imaging and other baseline information. No patients had local vascular invasion and portal vein invasion/resection or arterial resection. All enrolled patients did not receive neoadjuvant therapy before radical resection. The inclusion criteria included: 1) No arterial tumor contact (celiac axis, superior mesenteric artery, or common hepatic artery); 2) No tumor contact with the superior mesenteric vein or portal vein or ≤180° contact without vein contour irregularity; 3) Without any form of anti-tumor treatment before operation; 4) Patients with pancreatic ductal adenocarcinoma confirmed by histopathology. The exclusion criteria were as follows: 1) patients with non-R0 resection; 2) histopathologically confirmed patients with non-PDAC; 3) patients who died within 3 months after the operation; 4) patients whose informed consent was not signed or follow-up records were incomplete; 5) patients who had a history of other malignant tumors; 6) patients who received neoadjuvant chemotherapy; 7) Patients whose tumor invaded the celiac artery, superior mesenteric artery, and/or common hepatic artery. These patients were randomly assigned to the training group (218 patients) and the validation group (109 patients) at the ratio 2:1. This retrospective study had been approved by the Ethics Committee of the TJMUCIH.

### Histopathology Characteristics

Tumor tissues were collected during the operation with their pathological information, which include, vascular cancer embolus, capsule invasion, perineural invasion, tumor size, regional lymph node metastasis and pathological grade. Smoking index, alcohol consumption, diabetes, abdominal pain and body weight were also recorded. The Charson age comorbidity index (CACI) was calculated on the basis of the prospectively maintained institutional database, and 4 points were used to determine the cutoff value as described in published references (5). R0 resection was evaluated by two independent pathologists according to the statement of the International Pancreatic Surgery Group (27). When the distance between the tumor and the closest resection margin was greater than 1 mm, the resection margin (R) was defined as R0, otherwise it was defined as R1. All the patients were classified in accordance with the 8th edition of the American Joint Committee on Cancer.

### Tumor Biomarker and Laboratory Testing

Tumor markers (CA19-9, CEA and CA242), liver function indexes (ALB, ALP, TBIL, DBIL, gamma-glutamyltransferase and lactate dehydrogenase), systemic inflammatory parameters [neutrophils, platelets, lymphocytes, monocytes, NLR, PLR, LMR, prognostic nutrition index (PNI), neutrophil-to-ALB ratio (NAR) and platelet-to-ALB ratio (PAR)] and coagulation parameters [prothrombin time (PT), international normalised ratio (INR), activated partial thrombin time (APTT), Fbg, thrombin time (TT) and d-dimer DD] were collected 7 days before operation. Amongst them, liver function and systemic inflammatory parameters must be retested 1 week later the operation. Tumor markers should be re-examined 30 days later after operation. A total of 44 serum parameters, including preoperative and postoperative, were included in the current study. X-Tile software was used to automatically calculate the cutoff value of all the parameters and model scores (28).

### Follow Up of Patients

We followed the criteria of RECIST1.1, combined with imaging evaluation to judge the objective progress of tumor. All enrolled patients were continuously followed up, which was performed jointly by surgeons, physicians and radiologists. Patients were re-examined every 3 months in the first 2 years after operation, including imaging and tumor markers detection. If the results were stable, the interval of re-examination could be changed to once a year. Early recurrence was defined as recurrence within 1 year after radical resection as described before (5). OS was defined as the time between the date of surgery and the date of death due to any reason or the last follow-up. The median follow-up times of the training and validation groups were 41 months and 40 months, respectively; and the median survival times were 19 months and 20 months, respectively.

### Identification of Independent Risk Factors

To identify and verify the independent prognostic value of risk factors, we first performed univariate Cox regression analysis on 44 perioperative blood parameters using the R studio software (version 1.3.1056). Compared with the traditional stepwise regression method, LASSO regression punished the overfitting of data by constructing penalty coefficients, which reduced the interference of collinearity influencing factors between data. LASSO regression can actively select from a large number of variables with multicollinearity, reducing the possibility of data over fitting by constructing penalty coefficients (29). Then, 18 variables with *p* value less than 0.05 were further included in the LASSO (least absolute shrinkage and selection operator) regression analysis to screen out the variables related to early recurrence. During the LASSO regression, two analyses, the shrinkage coefficient diagram and the 10-fold cross-validation diagram were used to screen out the final perioperative serological factors that affect the early recurrence of PDAC patients with radical resection in the training cohort. The shrinkage coefficient diagram was used to determine the candidate variables that entered the model with the variation of λ value. As the value of λ increased, the greater the degree of model compressed, the less the number of candidate variables entered the model. The 10-fold cross-validation diagram was used to determine the lambda.min, which referred to the lambda value of the best model to control the decline of partial likelihood deviance. On this basis, 4 preoperative factors (platelets, Fbg, NLR and LMR) and 3 postoperative parameters (CA19-9, CEA and CA242) were screened out for the establishment of preoperative SICC and postoperative TMs scores.

### Construction and Validation of Nomograms

The preoperative SICC and postoperative TMs scores established by univariate Cox and LASSO regression as two variable parameters, together with the clinicopathological characteristics of patients, a total of 20 parameters, were included in univariate and multivariate Cox regression analysis, and further determined all independent risk factors associated with early recurrence and OS in patients. On this basis, the factors associated with the recurrence of PDAC patients after radical resection were identified as: preoperative SICC, postoperative TMs, CACI, vascular cancer embolus and adjuvant chemotherapy. The factors associated with overall survival were preoperative SICC, postoperative TMs, CACI, smoking index, vascular cancer embolus, and adjuvant chemotherapy. By taking into account all the variables, nomograms were established. The total points of each patient were calculated by the score model, and then divided into the high-score and low-score groups.

### Statistical Analysis

R Studio version 1.3.1056 was used for statistical analysis. Data comparison and correlation between groups were determined *via* nonparametric tests, chi-squared tests or Fisher accuracy as indicated. Univariate and multivariate Cox (“survival” package in R) and LASSO regression (“glmnet” package in R) were applied to analyse risk factors that affect prognosis. *p*-value and hazard ratio (HR) were calculated *via* Cox regression to determine the prognostic factors. The Kaplan-Meier method was used to compare survival between groups. The receiver operating characteristic (ROC) curve and the concordance index (C-index) were used to evaluate the performance of predictive model for recurrence or prognosis. Area under the curve (AUC) values were compared by Z-test. *p*<0.05 was considered statistically significant.

## Results

### Patient Characteristics

A total of 327 patients with radical resection were recruited for this study, and then randomly divided into the training group (218) and the validation group (109), with the same average age of 60 years. In the training group, 201 (92.2%) patients were younger than 70 years, and 124 (56.9%) patients were male, and 128 (58.7%) patients had a CACI greater than or equal to 4. In addition, 149 cases (68.3%) were located in the head and uncinate process of the pancreas, while 69 cases (31.7%) were in the body and tail. 108 patients (49.5%) were in stage I (21 patients in stage IA and 87 patients in stage IB), 93 patients (42.7%) were in stage II (30 patients in stage IIA and 63 patients in stage IIB), and 17 patients (7.8%) were in stage III due to the presence of more than 3 lymph node metastases. Based on the pathological features, there were 35 patients (16.0%) in T1-stage (tumor size ≤ 2), 134 patients (61.5%) in T2-stage (2 < tumor size ≤ 4cm), and 49 patients (22.5%) in T3-stage (tumor size > 4cm). 80 patients (36.7%) had lymph node metastasis that were confirmed by pathology, including 63 patients with N1-stage and 17 patients with N2-stage. Furthermore, 177 patients (81.2%) received gemcitabine-based systemic chemotherapy after resection (**Table 1**).

**Table 1:** Clinicopathological characteristics of patients in the training and validation groups.

Characteristics	Training (n)	Validation (n)	P-value
Total	218	109	–
Age, median (range)(years)	60 (39-80)	60 (36-84)	–
(<70 years vs. ≥70 years)	201 vs. 17	96 vs. 13	0.223
Gender (Male vs. Female)	124 vs. 94	73 vs. 36	0.079
CACI (≥4 vs. <4)	128 vs. 90	64 vs. 45	1.000
Smoking index (<400 vs. ≥400)	152 vs. 66	78 vs. 31	0.732
Alcohol consumption (No vs. Yes)	166 vs. 52	84 vs. 25	0.854
Family cancer history (No vs. Yes)	184 vs. 34	88 vs. 21	0.403
Diabetes (Absent vs. Present)	162 vs. 56	79 vs. 30	0.722
Pain (Present vs. Absent)	125 vs. 93	63 vs. 46	0.937
Weight loss (Present vs. Absent)	107 vs. 111	59 vs. 50	0.390
Tumor location (Head and Uncinate process vs. Body and Tail)	149 vs. 69	75 vs. 34	0.933
Differentiation (Poor vs. Well/Moderate)	147 vs. 71	60 vs. 49	0.028
Tumor size (≤4.0 cm vs. >4.0cm)	169 vs. 49	87 vs. 22	0.635
Regional lymph (N0 vs. N1 and N2)	138 vs. 80	77 vs. 32	0.187
Pathological stage (I and IIA vs. IIB and III)	138 vs. 80	77 vs. 32	0.187
Pancreatic capsule invasion (Present vs. Absent)	159 vs. 59	83 vs. 26	0.533
Perineural invasion (Present vs. Absent)	111 vs. 107	65 vs. 44	0.136
Vascular cancer embolus (Absent vs. Present)	185 vs. 33	93 vs. 16	0.913
Adjuvant chemotherapy (yes vs. no)	177 vs. 41	77 vs. 32	0.031

Similarly, we also analysed the constituent ratio of each clinicopathological feature in the validation group, and compared with that in the training group. As expected, most of the clinicopathological features did not differ statistically between the two groups, except the differentiation degree and the ratio of patients that received adjuvant chemotherapy (**Table 1**), suggesting an entirely acceptable homogeneity between these two cohorts.

### Identification of Parameters Related to Early Recurrence

**Figure 1:**
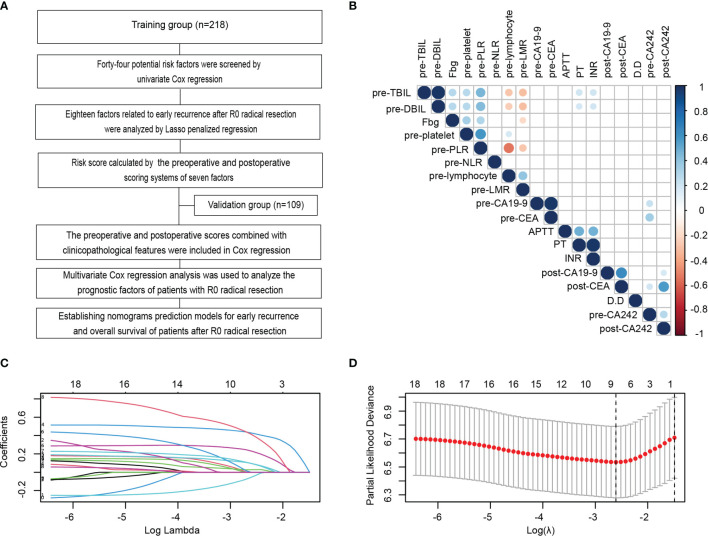
Identification and establishment of scoring systems related to serum indexes for early recurrence during the perioperative period. **(A)** Flowchart of the establishment of scoring systems and nomograms for the early recurrence and long-term prognosis of PDAC patients after radical resection in this study. **(B)** Correlation analysis amongst 18 variables with *p* values less than 0.05 in the univariate Cox analysis of the training cohort. **(C, D)** LASSO analysis of the 18 variables with *p* < 0.05 in the univariate Cox regression analysis to screen out the perioperative serological factors that affect the early recurrence of PDAC patients with radical resection in the training cohort. Shrinkage coefficient diagram was shown in **(C)**. Each curve represented the change track of each candidate variable coefficient. As the value of λ increased, the greater the degree of model compressed, the less the number of candidate variables entered the model. Ten-fold cross-validation diagram showed the determination of the best λ Value **(D)**.

On the basis of published studies and clinical experience, 44 blood parameters that might have been perceived as contributing to the postoperative early recurrence of PDAC patients were initially selected in the training group, and then evaluated using the Cox regression models (**Figure 1A**). In the univariate survival analysis, 18 of the 44 variables were statistically corelated with early recurrence of PDAC patients after radical resection (**Table 2**). We next analysed the correlation of these 18 variables, and determined that they were interrelated rather than independent influencing variables (**Figure 1B** and **Supplementary Table 1**).

**Table 2:** Univariate Cox regression analysis of 44 perioperative blood parameters in patients with PDAC after radical resection.

Parameters	Beta	HR (95% CI for HR)	Wald.Test	p.value
^a^ Pre-CA19-9 (<942.4 vs. ≥942.4)	0.652	1.920 (1.310-2.820)	11.10	<0.001
Pre-CEA (<8.03 vs. ≥8.03)	0.441	1.550 (1.050-2.300)	4.82	0.028
Pre-CA242 (<14.93 vs. ≥14.93)	0.407	1.500 (1.100-2.040)	6.72	0.010
^b^ Post-CA19-9 (<31.11 vs. ≥31.11)	1.110	3.030 (2.240-4.100)	51.40	<0.001
Post-CEA (<2.56 vs. ≥2.56)	0.517	1.680 (1.240-2.260)	11.50	<0.001
Post-CA242 (<11.68 vs. ≥11.68)	0.822	2.280 (1.680-3.080)	28.30	<0.001
Pre-neutrophils (<3.73 vs. ≥3.73)	0.272	1.310 (0.976-1.760)	3.24	0.072
Pre-lymphocytes (<1.4 vs. ≥1.4)	-0.334	0.716 (0.532-0.964)	4.85	0.028
Pre-monocytes (<0.43 vs. ≥0.43)	0.088	1.090 (0.812-1.470)	0.34	0.561
Pre-platelets (<329 vs. ≥329)	0.728	2.070 (1.400-3.070)	13.10	<0.001
Pre-ALB (<46.5 vs. ≥46.5)	0.231	1.260 (0.850-1.870)	1.33	0.250
Pre-NLR (<1.95 vs. ≥1.95)	0.628	1.870 (1.340-2.610)	13.70	<0.001
Pre-PLR (<191.96 vs. ≥191.96)	0.450	1.570 (1.150-2.140)	7.91	0.005
Pre-LMR (<4.7 vs. ≥4.7)	-0.359	0.699 (0.503-0.970)	4.59	0.032
Pre-PNI (<54.15 vs. ≥54.15)	0.113	1.120 (0.796-1.570)	0.42	0.518
Pre-NAR (<0.09 vs. ≥0.09)	0.141	1.150 (0.857-1.550)	0.87	0.350
Pre-PAR (<7.71 vs. ≥7.71)	0.042	1.040 (0.696-1.560)	0.04	0.839
Pre-ALP (<68 vs. ≥68)	0.182	1.200 (0.745-1.930)	0.56	0.454
Pre-TBIL (<382.1 vs. ≥382.1)	0.631	1.880 (1.110-3.200)	5.43	0.020
Pre-DBIL (<189.9 vs. ≥189.9)	0.541	1.720 (1.080-2.740)	5.16	0.023
Pre-GGT (<19 vs. ≥19)	0.169	1.180 (0.811-1.730)	0.76	0.382
Pre-LDH (<142 vs. ≥142)	0.542	1.720 (0.878-3.370)	2.50	0.114
Post-neutrophils (<6.54 vs. ≥6.54)	0.248	1.280 (0.940-1.750)	2.46	0.117
Post-lymphocytes (<1.39 vs. ≥1.39)	0.139	1.150 (0.853-1.550)	0.84	0.360
Post-monocytes (<0.67 vs. ≥0.67)	0.209	1.230 (0.912-1.670)	1.85	0.174
Post-platelets (<409 vs. ≥409)	0.241	1.270 (0.931-1.740)	2.28	0.131
Post-ALB (<33.3 vs. ≥33.3)	0.228	1.260 (0.901-1.750)	1.81	0.178
Post-NLR (<4.38 vs. ≥4.38)	0.152	1.160 (0.855-1.590)	0.93	0.334
Post-PLR (<327.94 vs. ≥327.94)	0.110	1.120 (0.807-1.550)	0.44	0.505
Post-LMR (<0.74 vs. ≥0.74)	0.733	2.080 (0.772-5.610)	2.10	0.147
Post-PNI (<39.40 vs. ≥39.40)	0.298	1.350 (0.955-1.900)	2.88	0.090
Post-NAR (<0.15 vs. ≥0.15)	0.296	1.340 (0.932-1.940)	2.51	0.113
Post-PAR (<14.49 vs. ≥14.49)	0.050	1.050 (0.713-1.550)	0.06	0.800
Post-ALP (<69 vs. ≥69)	0.567	1.760 (0.781-3.980)	1.87	0.172
Post-TBIL (<218.9 vs. ≥218.9)	0.597	1.820 (0.674-4.900)	1.40	0.238
Post-DBIL (<24.9 vs. ≥24.9)	0.204	1.230 (0.862-1.750)	1.29	0.256
Post-GGT (<68 vs. ≥68)	0.241	1.270 (0.902-1.800)	1.89	0.169
Post-LDH (<223.19 vs. ≥223.19)	0.149	1.160 (0.844-1.600)	0.84	0.360
PT (<11.1 vs. ≥11.1)	0.401	1.490 (1.110-2.010)	6.94	0.008
INR (<1.04 vs. ≥1.04)	0.392	1.480 (1.090-2.010)	6.24	0.013
APTT (<21.5 vs. ≥21.5)	0.611	1.840 (1.000-3.390)	3.84	0.049
Fbg (<3.851 vs. ≥3.851)	0.517	1.680 (1.240-2.270)	11.20	0.001
TT (<17.1 vs. ≥17.1)	0.349	1.420 (0.848-2.370)	1.77	0.184
D-D (<295.82 vs. ≥295.82)	0.359	1.430 (1.000-2.040)	3.90	0.048


^a^Pre-,means preoperative; ^b^Post-,means postoperative.

Compared with the traditional stepwise regression method, LASSO regression punished the over fitting of data by constructing penalty coefficient, which reduced the interference of collinearity influencing factors between data. Therefore, we used LASSO regression to further analyze these 18 variables. The shrinkage coefficient diagram may force and generate coefficients that are exactly 0 during operation. It is selected to retain non-zero variables in the model and generate a set of more relevant and interpretable prediction values to build the optimal model. As the value of λ increased, the greater the degree of model compressed, the less the number of candidate variables entered the model (**Figure 1C**). The 10-fold cross-validation diagram was used to determine the lambda.min, which referred to the lambda value of the best model to control the decline of partial likelihood deviance. The lambda.min was calculated as 0.07373468 (**Figure 1D**). At this time, the factors entering the model that significantly affected the early recurrence after radical resection only included 4 preoperative factors (platelets, Fbg, NLR and LMR) and 3 postoperative parameters (CA19-9, CEA and CA242). According to the screening variable coefficient, we established the preoperative and postoperative scoring formulas as follows:


Preoperative SICC score=0.414*platelet+0.096*Fbg+0.028*NLR−0.055*LMR

PostoperativeTMs score=0.428*CA19−9+0.097*CEA+0.028*NLR+0.232*CA242

Total score=Preoperative SICC score+ Postoperative TMs score

Based on these formulas, we calculated the preoperative SICC and postoperative TMs of each patient, and then used X-Tile to determine their best cutoff values for prediction of early recurrence. Eventually, the cutoff values of preoperative SICC and postoperative TMs for effectively predicting early recurrence of PDAC patients after radical resection were set as 0.4 and 0.6, respectively.

### ROC Curves of the Scoring Systems

**Figure 2:**
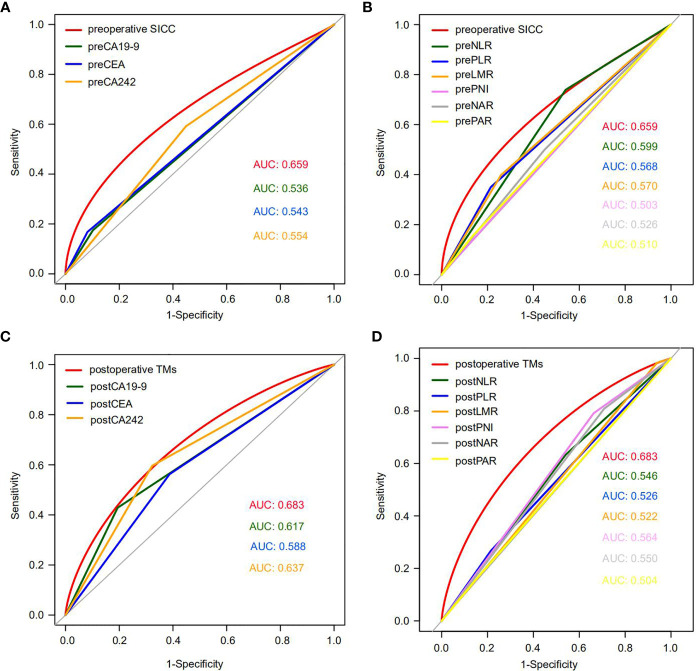
The ROC curves of preoperative SICC, postoperative TMs, tumor markers and six combinations of inflammatory factors in the training cohort. **(A)** Comparison of the ROC curves for early recurrence between preoperative SICC and preoperative tumor markers for PDAC patients who underwent radical resection. **(B)** Comparison of the ROC curves for early recurrence between preoperative SICC and preoperative six combinations of inflammatory markers. **(C)** Comparison of the ROC curves for early recurrence between postoperative TMs and postoperative tumor markers. **(D)** Comparison of the ROC curves for early recurrence between postoperative TMs and postoperative six combinations of inflammatory markers.

To evaluate the accuracy of our newly developed scoring systems in predicting early recurrence, we determined the ROC curves of our preoperative and postoperative scoring systems, as well as the currently used tumor markers (CA19-9, CEA, CA242), and the five independent inflammatory markers (neutrophils, platelets, lymphocytes, monocytes and ALB) or even their combinations (NLR, PLR, LMR, PNI, NAR and PAR) during the preoperative and postoperative period in the training group, and then compared their performance in predicting early recurrence of PDAC patients (**Table 3**). Encouragingly, our data indicated that the AUC of our preoperative SICC was significantly higher than those of CA19-9 (0.659 vs 0.536; *p*=0.006), CEA (0.659 vs 0.543; *p*=0.01) and CA242 (0.659 vs 0.554; *p*=0.044), as well as neutrophils (0.659 vs 0.552; *p*=0.010), platelets (0.659 vs 0.569; *p*=0.004), lymphocytes (0.659 vs 0.560; *p*=0.041), monocytes (0.659 vs 0.504; *p*<0.001) and ALB (0.659 vs 0.508; *p*=0.001), or even NLR (0.659 vs 0.599; *p*=0.032), PLR (0.659 vs 0.568; *p*=0.009), LMR (0.659 vs 0.570; *p*=0.047), PNI (0.659 vs 0.503; *p*=0.003), NAR (0.659 vs 0.526; *p*=0.010) and PAR (0.659 vs 0.510; *p*<0.001) (**Figures 2A, B** and **Table 3**), suggesting that the preoperative application of SICC was more accurate than the currently used tumor and inflammatory markers in predicting early recurrence.

**Table 3:** Comparison of the area under curve (AUC) of the blood scoring systems and inflammatory markers.

Parameters	pre^a^			post^b^		
	AUC (95%CI)	*p* value	*p* value^c^	AUC (95%CI)	*p* value	*p* value^d^
CA19-9	0.536 (0.491-0.582)	0.063	0.006	0.617 (0.558-0.677)	<0.001	0.424
CEA	0.543 (0.500-0.587)	0.030	0.010	0.588 (0.522-0.654)	0.005	0.120
CA242	0.554 (0.487-0.622)	0.059	0.044	0.637 (0.572-0.701)	<0.001	0.601
Neutrophils	0.552 (0.486-0.618)	0.063	0.010	0.546 (0.480-0.612)	0.345	0.006
Platelets	0.569 (0.526-0.613)	0.002	0.004	0.538 (0.477-0.599)	0.113	0.005
Lymphocytes	0.560 (0.497-0.624)	0.033	0.041	0.519 (0.452-0.586)	0.707	0.002
Monocyte	0.504 (0.437-0.571)	0.457	<0.001	0.527 (0.461-0.594)	0.211	0.001
Albumin	0.508 (0.460-0.556)	0.377	0.001	0.561 (0.498-0.623)	0.029	0.017
NLR	0.599 (0.536-0.663)	0.001	0.032	0.546 (0.480-0.612)	0.084	0.005
PLR	0.568 (0.509-0.627)	0.014	0.009	0.526 (0.469-0.583)	0.186	0.001
LMR	0.570 (0.498-0.625)	0.028	0.047	0.522 (0.496-0.549)	0.042	<0.001
PNI	0.503 (0.445-0.560)	0.466	0.003	0.564 (0.505-0.624)	0.017	0.018
NAR	0.526 (0.459-0.592)	0.227	0.010	0.550 (0.492-0.608)	0.043	0.005
PAR	0.510 (0.463-0.557)	0.340	<0.001	0.504 (0.454-0.554)	0.435	<0.001
Preoperative SICC	0.659 (0.586-0.733)	<0.001	–	–	–	0.633
Postoperative TMs	–	–	0.633	0.683 (0.608-0.758)	<0.001	–
Total score	0.719 (0.653-0.786)	<0.001	0.118	0.719 (0.653-0.786)	<0.001	0.083

^a^Indicates preoperative serum index.

^b^Indicates postoperative serum index.

^c^The AUC values between preoperative SICC and other inflammatory factors were compared by Z-test.

^d^The AUC values between postoperative TMs and other inflammatory factors were compared by Z-test.

Interestingly, compared with the preoperative SICC, after operation more attention should be given to the changes in tumor markers, which were more effective than that of the postoperative immune indexes for prediction of early recurrence (**Figures 2C, D** and **Table 3**). Although the AUC between the postoperative TMs (0.683) and the independent postoperative tumor markers (CA19-9, 0.617; CEA, 0.588; CA242, 0.637) was no statistical difference, postoperative TMs still provided the maximum AUC value in predicting early recurrence (**Figure 2C** and **Table 3**). In addition, no statistical difference was observed among the preoperative SICC, postoperative TMs and total scores for predicting early recurrence (**Table 3**), suggesting that our new scoring systems may help doctors evaluate the early recurrence of PDAC patients both at preoperative and postoperative stage.

### Prognostic Stratification Value of Serum Scoring Systems for Patients With Resectable PDAC

To further investigate the accuracy of the serum scoring systems in predicting the recurrence and long-term prognosis of patients with resectable PDAC, we performed univariate and multivariate Cox analysis in the training group to assess the efficacy of preoperative SICC and postoperative TMs score, as well as the clinical pathological variables for predicting the relapse-free survival (RFS) and overall survival (OS) of patients. In the univariate Cox analysis, preoperative SICC ≥ 0.4, postoperative TMs ≥ 0.6, CACI ≥ 4, lymph node metastasis, vascular cancer embolus, high pathological stage and no postoperative adjuvant chemotherapy were the risk factors for both recurrence and poor OS. In addition, poor pathological differentiation was also a risk factor for recurrence, but not for poor OS, while smoking index ≥ 400 was just the opposite. Moreover, incorporating the aforementioned variables into multivariate Cox analysis indicated that preoperative SICC ≥ 0.4, postoperative TMs ≥ 0.6, CACI ≥ 4, vascular cancer embolus and not receiving adjuvant chemotherapy were independent risk factors for both early recurrence and poor OS (**Table 4**).

**Table 4:** Univariate and multivariate analysis of recurrence and long-term prognosis in the training group.

Characteristics	Relapse-free survival		Overall survival	
	HR (95%CI)	p-value	HR (95%CI)	p-value
**Univariate analysis**				
Preoperative SICC (≥0.4 vs. <0.4)	6.540 (2.800-15.300)	<0.001	5.770 (2.490-13.400)	<0.001
Postoperative TMs (≥0.6 vs. <0.6)	4.090 (2.460-6.830)	<0.001	5.620 (3.350-9.440)	<0.001
Gender (Male vs. Female)	0.855 (0.630-1.160)	0.314	0.733 (0.535-1.00)	0.052
Age (≥70 years vs. <70 years)	0.962 (0.546-1.700)	0.893	0.979 (0.543-1.760)	0.943
CACI (≥4 vs. <4)	1.700 (1.240-2.330)	<0.001	2.150 (1.540-3.000)	<0.001
Smoking Index (≥400 vs. <400)	1.270 (0.913-1.760)	0.157	1.490 (1.080-2.070)	0.016
Alcohol consumption (yes vs. no)	1.090 (0.764-1.560)	0.630	1.290 (0.909-1.840)	0.153
Diabetes (yes vs. no)	1.090 (0.777-1.540)	0.610	0.960 (0.672-1.370)	0.822
Abdominal pain (yes vs. no)	1.270 (0.929-1.720)	0.135	1.210 (0.880-1.660)	0.244
Weight loss (yes vs. no)	0.963 (0.712-1.300)	0.806	1.120 (0.824-1.530)	0.466
Operation procedure (PD vs. DP)	0.885 (0.644-1.220)	0.453	0.830 (0.600-1.150)	0.259
Differentiation (Poor vs. well/moderate)	1.420 (1.020-1.970)	0.037	1.250 (0.892-1.740)	0.196
Tumor size (>4.0cm versus ≤4.0 cm)	1.100 (0.765-1.580)	0.611	1.360 (0.949-1.940)	0.094
lymph node (N) (0 vs. 1 and 2)	1.280 (1.010-1.620)	0.043	1.370 (1.090-1.740)	0.007
Positive lymph node ratio (>0.2 vs. ≤0.2)	1.380 (0.934-2.050)	0.105	1.550 (1.050-2.290)	0.028
Pancreatic capsule invasion (Present vs. Absent)	0.963 (0.689-1.340)	0.824	1.070 (0.753-1.510)	0.721
Perineural invasion (Present vs. Absent)	1.050 (0.776-1.420)	0.758	1.080 (0.794-1.470)	0.626
Vascular cancer embolus (Present vs. Absent)	1.660 (1.100-2.500)	0.015	1.510 (1.010-2.270)	0.044
Pathological stage (I/IIA vs. IIB/III)	1.390 (1.010-1.900)	0.041	1.420 (1.030-1.940)	0.031
Adjuvant chemotherapy (yes vs. no)	1.710 (1.160-2.540)	0.007	2.410 (1.650-3.530)	<0.001
**Multivariate analysis**				
Preoperative SICC (≥0.4 vs. <0.4)	4.646 (1.887-11.438)	<0.001	2.834 (1.161-6.915)	0.022
Postoperative TMs (≥0.6 vs. <0.6)	3.205 (1.891-5.434)	<0.001	3.821 (2.187-6.676)	<0.001
CACI (≥4 vs. <4)	1.414 (1.017-1.966)	0.039	1.630 (1.140-2.329)	0.007
Smoking Index (≥400 vs. <400)	–	–	1.553 (1.110-2.172)	0.010
Vascular cancer embolus (Present vs. Absent)	1.702 (1.080-2.684)	0.022	1.709 (1.114-2.624)	0.014
Adjuvant chemotherapy (yes vs. no)	1.551 (1.026-2.347)	0.038	1.745 (1.162-2.621)	0.007

**Figure 3:**
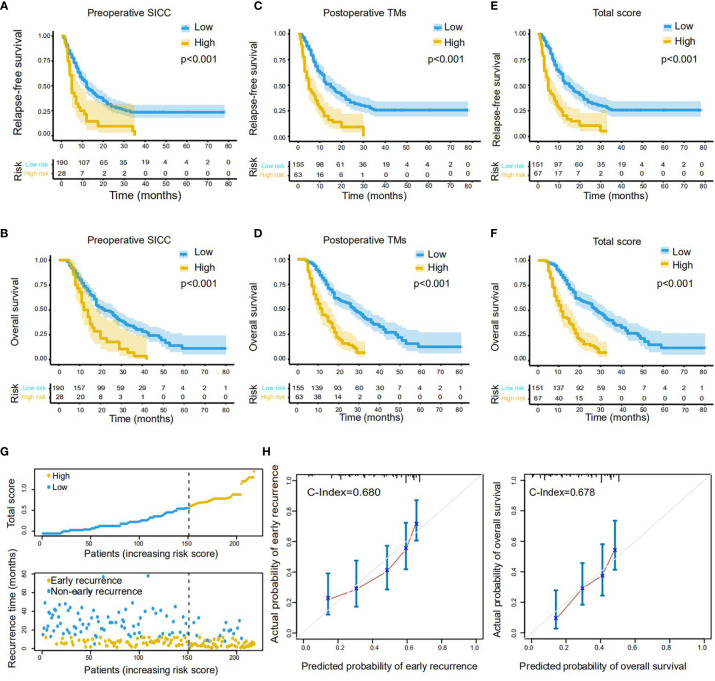
Performance of the scoring systems on RFS and OS prediction in the training cohort. **(A, B)** The Kaplan-Meier survival curves of RFS **(A)** and OS **(B)** for PDAC patients with low or high preoperative SICC (Value_cutoff_ = 0.4). **(C, D)** The Kaplan-Meier survival curves of RFS **(C)** and OS **(D)** for patients with low or high postoperative TMs (Value_cutoff_ = 0.6). **(E, F)** The Kaplan-Meier survival curves of RFS **(E)** and OS **(F)** for patients with low or high total score (Value_cutoff_ = 0.6). **(G)** Distribution of the total score and related recurrence data in the training cohort. **(H)** Calibration plot for the internal validation of the total score on RFS (left) and OS (right) evaluation. The Y-axis represents the actual rate. The X-axis represents the predicted rate. Each cutoff value was calculated *via* X-Tile.

Notably, Kaplan-Meier survival analysis demonstrated that patients with preoperative SICC ≥ 0.4 displayed much lower survival times than that with preoperative SICC < 0.4 both in RFS (median survival: 5 vs 12 months) and OS (median survival: 12 vs 22 months) (**Figures 3A, B**). Similarly, patients with postoperative TMs ≥ 0.6 or total score ≥ 0.6 also represented the poor RFS (median survival: 5 vs 15 months in both scores) and OS (median survival: 12 vs 28 months in postoperative TMs, 11 vs 28 months in total score) when compared to the patients with lower score (**Figures 3C–F**). Furthermore, in accordance with patients’ total score and recurrence prognosis distribution (**Figure 3G**), and by comparing with the actual recurrence rate and survival rate, the total score predicted the early recurrence and long-term survival of patients with C-index values of 0.680 and 0.678, respectively (**Figure 3H**), suggesting a high efficiency of this scoring system.

To confirmed these data, we next performed the same analysis in the validation group with a cohort of 109 patients. As expected, all of our newly developed preoperative SICC, postoperative TMs and total scores could effectively evaluate the RFS and OS of the patients in the validation group (**Supplementary Figures 1A–F**). Meanwhile, the total score was very consistent in predicting the early recurrence and long-term survival of patients over these two cohorts (**Supplementary Figures 1G, H**). Altogether, these results demonstrated that our scoring systems may be helpful for effectively identifying PDAC patients with rapid recurrence or metastasis after radical resection.

### Construction and Verification of Nomograms for Early Recurrence and Long-Term Prognosis Prediction

**Figure 4:**
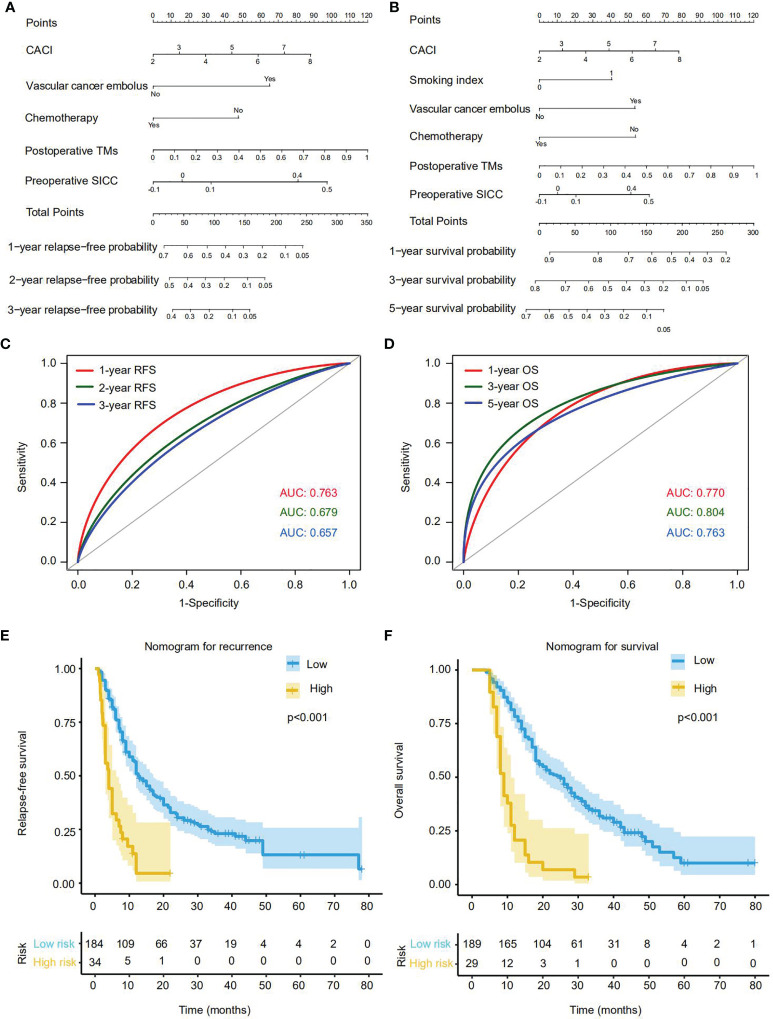
Establishment and effectiveness evaluation of nomograms for predicting the recurrence or OS rate of PDAC patients with radical resection in the training cohort. **(A)** Nomogram for predicting 1-, 2- and 3-year recurrence. **(B)** Nomogram for predicting 1-, 3- and 5-year OS. **(C)** The ROC curves and AUC values of the nomogram for 1-, 2- and 3-year recurrence predictions. **(D)** The ROC curves and AUC values of the nomogram for 1-, 3- and 5-year OS predictions. **(E, F)** The Kaplan-Meier survival curves of RFS **(E)** and OS **(F)** for patients with low or high score according to the nomograms (cutoff_recurrence_ = 191; cutoff_survival_ = 185). Each cutoff value was calculated *via* X-Tile.

On the basis of the aforementioned multivariate Cox regression model, we then established two independent nomograms for predicting recurrence at 1, 2 or 3 years and survival at 1, 3 or 5 years, respectively. The factors for predicting recurrence and survival both included CACI, vascular cancer embolus, adjuvant chemotherapy, preoperative SICC and postoperative TMs (**Figures 4A, B**). Besides, the smoking index was also involved in the survival prediction (**Figure 4B**). To assess the efficacy of these two nomograms in recurrence and survival prediction, we determined their ROC curves for RFS and OS evaluation in the training cohort. The results indicated that AUC values of 1-, 2- and 3-years RFS were 0.763, 0.679 and 0.657 (**Figure 4C**), while the 1-, 3- and 5-year OS were 0.770, 0.804 and 0.763, respectively (**Figure 4D**), representing a great performance for these two nomograms in predicting recurrence and survival of PDAC after radical resection.

According to the nomograms, we next calculated the patient risk score in recurrence and prognosis prediction. By using the X-tile plot, we finally determined the cutoff values of recurrence and survival as 191 and 185, respectively, and then divided the training cohort into high-risk and low-risk groups. As expected, in contrast with the low-risk group, high-risk group were more likely to suffer from relapse (*p*<0.001), and displayed much worse OS rates (*p*<0.001) (**Figures 4E, F**).

Simultaneously, we also performed the same analysis in the validation cohort and obtained the consistent results that our newly established nomograms could effectively predict the recurrence and survival of PDAC patients with radical resection. (**Supplementary Figure 2A-D**). Thus, we have developed a series of accessible scoring systems and nomograms that display high potential to help clinicians identify resectable PDAC patients with high risk of early recurrence and metastasis for precision medicine and early intervention.

## Discussion

Clinically, some PDAC patients who underwent radical resection experienced rapid recurrence and metastasis after operation, but may benefit from neoadjuvant chemotherapy (5). By setting the time threshold for early recurrence at 12 months, approximately 53.8% (176/327) patients in our cohorts experienced early recurrence after radical resection. Thus, an effective and accessible evaluation system for accurately identifying these patients is urgently needed in clinic, but is currently lacked. Current evaluation systems just consider limited parameters 1 month or more before or after surgery, whilst disregarding the indicators during the perioperative period, which in fact, is more crucial for the prognosis prediction of resectable PDAC (25). In this study, we recruited 327 PDAC patients with radical resection from our centre and randomly divided into two cohorts (the training and validation groups). In the training cohort, 44 perioperative serum parameters during the perioperative period, including systemic inflammatory system parameters, coagulation system parameters, tumor markers and other parameters, were systematically analysed to determine factors that may affect early recurrence. After univariate Cox screening, variables with *p* value less than 0.05 were included in the LASSO regression model to undergo a more rigorous screening process. Our data indicated that 4 preoperative parameters (platelets, Fbg, NLR and LMR) and 3 postoperative parameters (CA19-9, CEA and CA242) were important risk factors for early recurrence of PDAC patients after radical resection, which was very consistent in the validation cohort. These findings suggested that more attention should be given to coagulation cascades and systemic inflammatory response before operation and tumor markers changes after operation.

The coagulation and immune systems play important roles in the occurrence and development of tumors (30, 31). The interaction between platelets and tumor cells is a prerequisite for tumor blood metastasis, which could help tumor cells escape from immunosurveillance, and facilitates proliferation and colonisation (32, 33). It is well known that Fbg is closely related to the development of inflammation-driven malignant tumors through promoting proliferation, angiogenesis and the expression of key inflammatory mediators (30). A meta-analysis of 15371 patients showed that an increase in plasma Fbg before treatment was significantly associated with a decrease in the survival of patients with solid tumors (34). Lots of studies have confirmed that many systemic immune factors and their combinations, such as neutrophils (35), lymphocytes (36), monocytes (37), platelets (38), ALB (39), NLR (40), PLR (41), LMR (42), NAR (43) and PAR (44) could predict the prognosis of patients. Amongst them, neutrophils and monocytes are regarded as risk factors for the prognosis, while lymphocytes are considered as protective factors for tumor patients (37, 45), which is consistent with our findings.

CA19-9 is well-known as the preferred biomarker that is recommended in NCCN guidelines for the clinical management of PDAC (46). However, it should be noted that the sensitivity of CA19-9 in patients with early PDAC is much lower than that with late PDAC (47). Besides, CEA and CA242 are other two commonly used tumor markers in clinic. Notably, the patients with positive expression of more than two of these tumor markers have significantly shorter survival time than that with only one or no expression (19). Interestingly, a retrospective cohort study reported that patients with elevated preoperative CEA that returned to normal after colon cancer resection did not exhibit a higher risk of recurrence in contrast with normal preoperative CEA, suggesting that preoperative CEA was not a risk factor for postoperative recurrence. Instead, patients with elevated postoperative CEA were more prone to recurrence within 1 year after operation (48). This study further supports our results that postoperative but not preoperative tumor markers were risk factors for early recurrence of PDAC patients who underwent radical resection. That would mean more attention should be given to the postoperative tumor markers for monitoring the prognosis of resectable PDAC patients.

All of the parameters involved in this study are necessary inspection indicators for PDAC patients during the perioperative period. Therefore, our newly developed evaluation systems are very accessible, and will not increase the financial burden of patients. However, it should be noted that our study still has several limitations. Firstly, although huge number of parameters were considered in the beginning, just limited variables were finally involved in the evaluation models. Secondly, this study was a single-centre retrospective study, so the representativeness of these systems is limited, and need to be further investigate. In the future, we will continue to improve these scoring systems and nomograms by including more indicators and expanding the patient cohort to multi-centre.

In conclusion, we established preoperative SICC and postoperative TMs scoring models for effectively predicting early recurrence and prognosis of PDAC patients after radical resection, whose scores were identified as independent risk factors for recurrence and long-term survival in our cohorts. Moreover, on the basis of these scoring systems, we further constructed nomogram models for the accurate evaluation of the RFS and OS of PDAC patients who underwent radical resection. Thus, our study has provided a series of effective evaluation systems that may help clinicians identify PDAC patients with high risk of early recurrence and metastasis for individualised treatment.

## Data Availability Statement

The raw data supporting the conclusions of this article will be made available by the authors, without undue reservation.

## Ethics Statement

The studies involving human participants were reviewed and approved by Tianjin Medical University Cancer Institute and Hospital. The patients/participants provided their written informed consent to participate in this study.

## Author Contributions

SL: conceptualization, methodology, data curation, formal analysis, investigation, validation, visualization, writing-original draft. GZ: conceptualization, methodology, data curation, formal analysis, investigation, writing-original draft. YL: methodology, data curation, formal analysis, validation, investigation. TZ: resources, data curation, formal analysis. CG: resources, data curation, formal analysis. WL: data curation, formal analysis. YP: methodology, formal analysis, validation. CH: conceptualization, formal analysis, supervision, funding acquisition. YC: writing-review and editing. AC: conceptualization, formal analysis, investigation, supervision, project administration, funding acquisition, writing-review and editing. JH: conceptualization, resources, supervision, funding acquisition, writing-review and editing.

## Funding

This work was supported by the National Key Research and Development Program of China (2021YFA1201100), the National Natural Science Foundation of China (grants 82030092, 81720108028, 82072657, 81802432, 82072716, 81802433, 82072659, 82173295, 81871968, 81871978, 82072691 and 82103006), the programs of Tianjin Prominent Talents, Tianjin Eminent Scholars, Tianjin Natural Science Foundation (18JCJQJC47800,19JCJQJC63100, 19JCYBJC26200 and 20JCQNJC01330).

## Conflict of Interest

The authors declare that the research was conducted in the absence of any commercial or financial relationships that could be construed as a potential conflict of interest.

## Publisher’s Note

All claims expressed in this article are solely those of the authors and do not necessarily represent those of their affiliated organizations, or those of the publisher, the editors and the reviewers. Any product that may be evaluated in this article, or claim that may be made by its manufacturer, is not guaranteed or endorsed by the publisher.

## Supplementary Material

Supplementary Figure 1 Verification of the performance of preoperative SICC and postoperative TMs scoring systems on RFS and OS evaluation in the validation cohort. **(A-B)** The Kaplan-Meier survival curves of RFS **(A)** and OS **(B)** for PDAC patients with low or high preoperative SICC (Value_cutoff_ = 0.4). **(C, D)** The Kaplan-Meier survival curves of RFS **(C)** and OS **(D)** for patients with low or high postoperative TMs (Value_cutoff_ = 0.6). **(E, F)** The Kaplan-Meier survival curves of RFS **(E)** and OS **(F)** for patients with low or high total score (Value_cutoff_ = 0.6). **(G)** Distribution of the total score and related recurrence data in the validation cohort. **(H)** Calibration plot for the external validation of the total score on RFS (left) and OS (right) evaluation. The Y-axis represents the actual rate. The X-axis represents the predicted rate. Each cutoff value was calculated *via* X-Tile.

Supplementary Figure 2 Verification of the nomograms for predicting the recurrence or OS rate of PDAC patients with radical resection in the validation cohort. **(A)** The ROC curves and AUC values of the nomogram for 1-, 2- and 3-year recurrence predictions in the validation cohort. **(B)** The ROC curves and AUC values of the nomogram for 1-, 3- and 5-year OS predictions in the validation cohort. **(C, D)** The Kaplan-Meier survival curves of RFS **(C)** and OS **(D)** for patients of the validation cohort with low or high score according to the nomograms (cutoff_recurrence_ = 191; cutoff_survival_ = 185). Each cutoff value was calculated *via* X-Tile.

## Abbreviations

AJCC, American joint committee on cancer; AUC, area under the curve; CA19-9, carbohydrate antigen 19-9; CA242, carbohydrate antigen 242; CACI, Charson age comorbidity index; CEA, carcinoembryonic antigen; CI, confidence interval; RFS, relapse-free survival; HR, hazard ratio; LASSO, least absolute shrinkage and selection operator; LMR, lymphocyte to monocyte ratio; NAR, neutrophil to albumin ratio; NLR, neutrophil to lymphocyte ratio; OS, overall survival; PAR, platelet to albumin ratio; PDAC, pancreatic ductal adenocarcinoma; PLR, platelet to lymphocyte ratio; PNI, prognostic nutrition index ROC, receiver operating characteristic; SICC, Systemic immune coagulation cascade; TMs, Tumor markers.
