# Supplementary figures and images for: Perioperative Serum Scoring Systems Predict Early Recurrence and Poor Prognosis of Resectable Pancreatic Cancer
Source: Front Oncol. 2022 Feb 21;12:841819. doi: 10.3389/fonc.2022.841819 (PMC8900727; doi:10.3389/fonc.2022.841819)

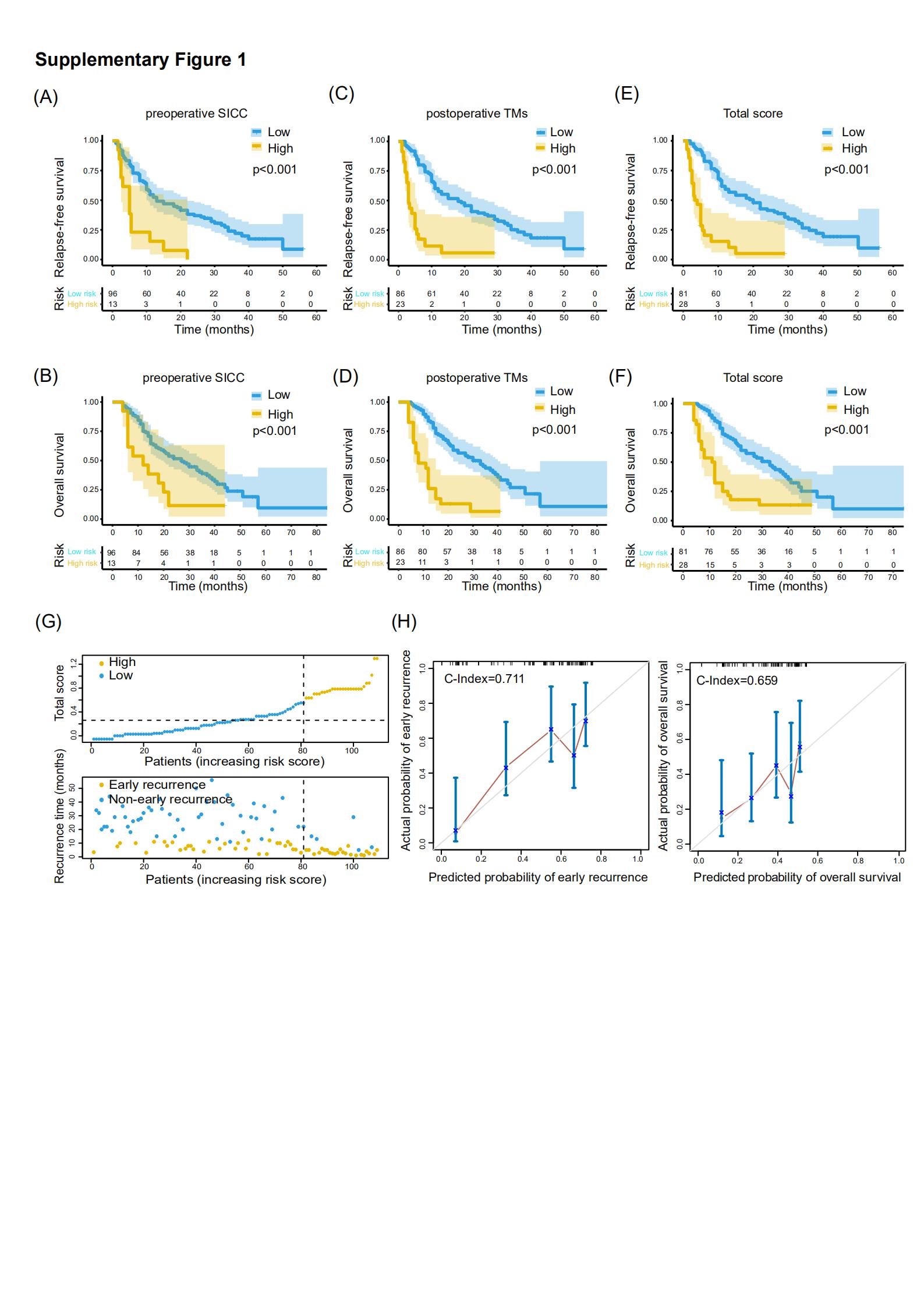

Supplement: Supplementary Figure 1 — Verification of the performance of preoperative SICC and postoperative TMs scoring systems on RFS and OS evaluation in the validation cohort. (A-B) The Kaplan-Meier survival curves of RFS (A) and OS (B) for PDAC patients with low or high preoperative SICC (Valuecutoff = 0.4). (C, D) The Kaplan-Meier survival curves of RFS (C) and OS (D) for patients with low or high postoperative TMs (Valuecutoff = 0.6). (E, F) The Kaplan-Meier survival curves of RFS (E) and OS (F) for patients with low or high total score (Valuecutoff = 0.6). (G) Distribution of the total score and related recurrence data in the validation cohort. (H) Calibration plot for the external validation of the total score on RFS (left) and OS (right) evaluation. The Y-axis represents the actual rate. The X-axis represents the predicted rate. Each cutoff value was calculated via X-Tile. [file Image_1.jpeg]

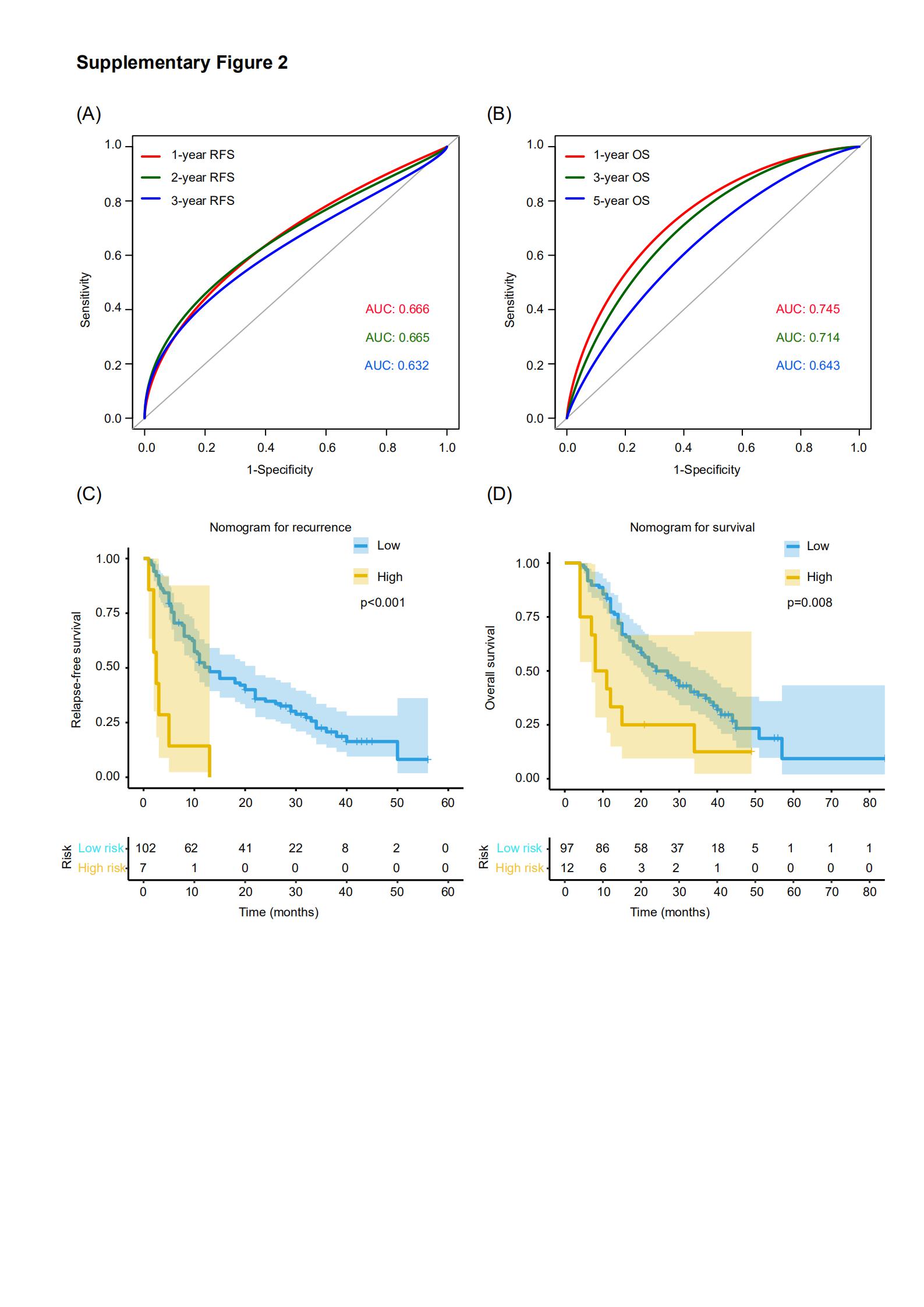

Supplement: Supplementary Figure 2 — Verification of the nomograms for predicting the recurrence or OS rate of PDAC patients with radical resection in the validation cohort. (A) The ROC curves and AUC values of the nomogram for 1-, 2- and 3-year recurrence predictions in the validation cohort. (B) The ROC curves and AUC values of the nomogram for 1-, 3- and 5-year OS predictions in the validation cohort. (C, D) The Kaplan-Meier survival curves of RFS (C) and OS (D) for patients of the validation cohort with low or high score according to the nomograms (cutoffrecurrence = 191; cutoffsurvival = 185). Each cutoff value was calculated via X-Tile. [file Image_2.jpeg]
